# The Sinus That Breeds Fungus: Subcutaneous Zygomycosis Caused by *Basidiobolus ranarum* at the Injection Site

**DOI:** 10.1155/2013/534192

**Published:** 2013-11-19

**Authors:** S. T. Jayanth, P. Gaikwad, M. Promila, J. C. Muthusami

**Affiliations:** ^1^Department of General Surgery, Christian Medical College, Vellore, India; ^2^Department of Microbiology, Christian Medical College, Vellore, India

## Abstract

Subcutaneous zygomycosis is caused by *Basidiobolus ranarum* which is endemic in India. We report a case of a housewife who presented with a persistent discharging sinus from the right gluteal region subsequent to an intramuscular injection which was refractory to empirical antituberculous therapy. She underwent an excision of the sinus tract, the culture of which yielded *B. ranarum*. The wound improved with oral potassium iodide.

## 1. Introduction

*Basidiobolus ranarum* is a saprophytic fungus present in soil, decaying fruit, and vegetable matter as well as in the gut of amphibians and reptiles. It can cause a variety of clinical manifestations including subcutaneous zygomycosis, gastrointestinal zygomycosis, and occasionally an acute systemic illness similar to that caused by the Mucorales [1]. Subcutaneous zygomycosis is the commonest presentation with cases reported from many tropical countries including India [2–4]. The present report deals with subcutaneous zygomycosis in a housewife residing in Chhattisgarh, Central India.

## 2. Case Report

A 58-year-old female, from Chhattisgarh, Central India, presented with a persistent discharging sinus from the right gluteal region for two years. She had previously received an intramuscular injection in the right gluteal region at another centre for fever and back pain. Subsequently she developed multiple abscesses over the right gluteal region that required incision and drainage on two occasions. She had been treated presumptively for tuberculosis with a 6-month course of antituberculous drugs (two months of Isoniazid, Rifampicin, Pyrazinamide, and Ethambutol followed by four months of Isoniazid and Rifampicin) with no apparent clinical improvement. 

On examination, there was a subcutaneous, nontender, indurated swelling in the right gluteal region with a single sinus opening with serous and nonfoul smelling pus discharge. Systemic examination was normal. Her laboratory values were hemoglobin 11.6 g%, white cell count 7000/mm^3^, platelets—2,50,000/mm^3^, fasting blood sugar 90 mg%, postprandial blood sugar 130 mg%, serum Creatinine 1.0 mg%, chest X-ray normal, and erythrocyte sedimentation rate of 15 mm at one hour.

**Figure 1:**
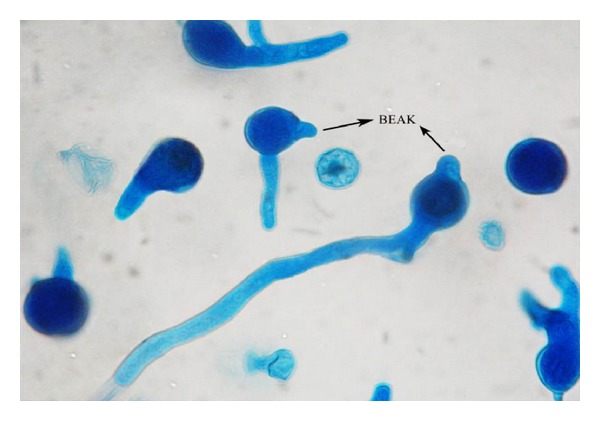
Lactophenol cotton blue preparation of *Basidiobolus ranarum*.

**Figure 2:**
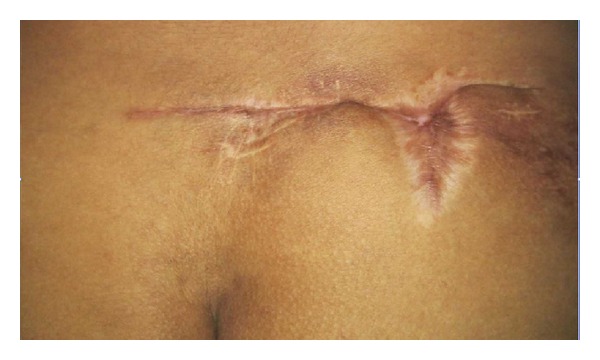
Completely healed wound at one-year follow-up.

A provisional diagnosis of a soft tissue infection due to atypical mycobacteria was made. The sinus tract was excised and a portion of the specimen was sent for histopathological as well as mycological examinations. Haematoxylin and eosin stained sections revealed inflammatory granulation tissue and foreign body granulomas with necrosis in the sinus tract. Two days postoperatively a blackish discoloration of the edge of the surgical wound was noted and was debrided. A calcofluor white preparation of the debrided tissue revealed broad, irregular, and aseptate hyphae. Culture on Sabouraud's dextrose agar showed a creamy white, waxy, and glabrous colony with many radial folds. On performing lactophenol cotton blue wet mount of the fungus, aseptate hyphae, and numerous globose, smooth walled zygospores with characteristic conjugation beaks were observed (Figure 1). The fungus was identified as *Basidiobolus ranarum*. There was an excellent response to oral potassium iodide in our patient with complete resolution in six months (Figure 2). 

## 3. Discussion

A localized subcutaneous and predominantly tropical mycosis is characterized by chronic woody swelling of the affected tissue. Basidiobolomycosis is the most common form of zygomycosis and is endemic in southern India [3–5]. Usually there are no predisposing factors (including the present case), though traumatic implantation from intramuscular injection use is probably the mode of entry as in other subcutaneous mycoses [5]. In the past, clinical isolates of *Basidiobolus* were classified as *B. ranarum, B. meristosporus,* and *B. haptosporus, *but recent taxonomic studies based on antigenic analysis, isoenzyme banding, and restriction enzyme analysis of rDNA indicate that all human pathogens belong to *B. ranarum*. 

Histologically, basidiobolomycosis is associated with eosinophilic infiltration. This has been postulated to be due to a predominant Th2 type of immune response with release of the cytokines IL-4 and IL-10 which in turn are helpful in recruiting eosinophils to the affected site [6]. Most patients with basidiobolomycosis respond very well to oral potassium iodide therapy as also to azoles particularly itraconazole [7, 8]. Treatment with Amphotericin B has given unsatisfactory results, with some strains even showing *in vitro* resistance to this drug [9]. This underscores the importance of recognizing and suspecting rare fungal infections and atypical mycobacterial infections when there is a persistent sinus beyond 6 weeks following abscess drainage. If there are obvious fungal colonies on the wound, then the diagnosis is evident. However, there are no other features to clinically differentiate atypical mycobacteria from basidiobolomycosis. Tissue culture confirms the diagnosis and needs appropriate treatment.

## 4. Conclusion

Soft tissue infections following iatrogenic causes are common. But subcutaneous zygomycosis is a rare cause of soft tissue infection and should be suspected in chronic nonhealing sinuses, abscesses, and atypical swellings following traumatic implantation. It has to be considered as a differential diagnosis in such refractory cases. Blackish discoloration when seen raises the level of suspicion. This can be effectively treated with oral potassium iodide for six months.
